# Extramural Venous Invasion and Tumor Deposit at Diffusion-weighted MRI in Patients after Neoadjuvant Treatment for Rectal Cancer

**DOI:** 10.1148/radiol.230079

**Published:** 2023-08-15

**Authors:** Tae-Hyung Kim, Canan Firat, Hannah M. Thompson, Natalie Gangai, Junting Zheng, Marinela Capanu, David D. B. Bates, Viktoriya Paroder, Julio García-Aguilar, Jinru Shia, Marc J. Gollub, Natally Horvat

**Affiliations:** From the Departments of Radiology (T.H.K., N.G., D.D.B.B., V.P., M.J.G., N.H.), Pathology (C.F., J.S.), Surgery (H.M.T., J.G.A.), and Epidemiology and Biostatistics (J.Z., M.C.), Memorial Sloan-Kettering Cancer Center, 1275 York Ave, Box 29, New York, NY 10065.

## Abstract

**Background:**

Diffusion-weighted (DW) imaging is useful in detecting tumor in the primary tumor bed in locally advanced rectal cancer (LARC) after neoadjuvant therapy, but its value in detecting extramural venous invasion (EMVI) and tumor deposit is not well validated.

**Purpose:**

To evaluate diagnostic accuracy and association with patient prognosis of viable EMVI and tumor deposit on DW images in patients with LARC after neoadjuvant therapy using whole-mount pathology specimens.

**Materials and Methods:**

This retrospective study included patients who underwent neoadjuvant therapy and surgery from 2018 to 2021. Innovative five-point Likert scale was used by two radiologists to independently evaluate the likelihood of viable EMVI and tumor deposit on restaging DW MRI scans in four axial quadrants (12 to 3 o’clock, 3 to 6 o’clock, 6 to 9 o’clock, and 9 to 12 o’clock). Diagnostic accuracy was assessed at both the per-quadrant and per-patient level, with whole-mount pathology as the reference standard. Weighted κ values for interreader agreement and Cox regression models for disease-free survival and overall survival analyses were used.

**Results:**

A total of 117 patients (mean age, 56 years ± 12 [SD]; 70 male, 47 female) were included. Pathologically proven viable EMVI and tumor deposit was detected in 29 of 117 patients (25%) and in 44 of 468 quadrants (9.4%). Per-quadrant analyses showed an area under the receiver operating characteristics curve of 0.75 (95% CI: 0.68, 0.83), with sensitivity and specificity of 55% and 96%, respectively. Good interreader agreement was observed between the radiologists (κ = 0.62). Per-patient analysis showed sensitivity and specificity of 62% and 93%, respectively. The presence of EMVI and tumor deposit on restaging DW MRI scans was associated with worse disease-free survival (hazard ratio [HR], 5.6; 95% CI: 2.4, 13.3) and overall survival (HR, 8.9; 95% CI: 1.6, 48.5).

**Conclusion:**

DW imaging using the five-point Likert scale showed high specificity and moderate sensitivity in the detection of viable extramural venous invasion and tumor deposits in LARC after neoadjuvant therapy, and its presence on restaging DW MRI scans is associated with worse prognosis.

Published under a CC BY 4.0 license.

*Supplemental material is available for this article.*

See also the editorial by Méndez and Ayuso in this issue.

## Graphical abstract

**Figure:**
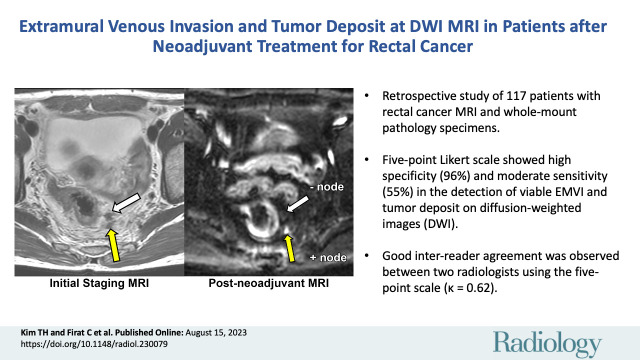

**Summary**

A five-point scale showed high specificity and moderate sensitivity in the detection of viable extramural venous invasion and tumor deposit on diffusion-weighted images and served to predict worse prognosis in patients with locally advanced rectal cancer after neoadjuvant therapy.

**Key Results**

■ In this retrospective study of 117 patients with rectal adenocarcinoma and available whole-mount pathology specimens, a five-point Likert scale showed high specificity (96%) and moderate sensitivity (55%) in the detection of viable extramural venous invasion (EMVI) and tumor deposit on diffusion-weighted (DW) images.
■ Good interreader agreement was observed between two radiologists using the five-point Likert scale (κ = 0.62).
■ Patients with EMVI and tumor deposit at DW imaging on postneoadjuvant MRI scans had shorter disease-free survival (hazard ratio [HR] = 5.6) and overall survival (HR = 8.9) than those without EMVI and tumor deposit, similar to patients with disease-free survival (HR = 9.4) and overall survival (HR = 7.9) at pathology.

## Introduction

Aside from the primary rectal tumor and lymph nodes, extramural venous invasion (EMVI) and tumor deposit are increasingly recognized on MRI scans as poor prognostic factors for patients with locally advanced rectal cancer (LARC). Lord et al (1) reported that the combination of baseline EMVI and tumor deposit on MRI scans was associated with poor overall survival and a hazard ratio of 2.07 and was the only prognosticator related to distant recurrence. Schaap et al (2) observed a higher rate of distant metastases in patients with baseline EMVI and tumor deposit on MRI scans compared with patients without.

Previous studies have reported that MRI has a sensitivity and specificity of 61% and 87%, respectively, for detection of EMVI (3) and of 91% and 68%, respectively, for detection of tumor deposit (4). However, studies such as these tend to perform per-patient analysis with a lack of point-by-point correlation between MRI and pathology results, which may result in overestimation of the performance of MRI. For example, EMVI detected on the right side of the tumor at MRI (hence MRI was positive for EMVI) and EMVI detected on the left side of tumor (hence pathology was positive for EMVI) may be scored as true positive at per-patient analysis. While a few studies have used whole-mount pathology as the reference standard to correlate EMVI on T2-weighted MRI scans (5,6), it can be difficult to differentiate between these features and viable tumor and fibrosis after neoadjuvant therapy.

Diffusion-weighted (DW) imaging with apparent diffusion coefficient mapping is a widely used MRI approach that adds value to T2-weighted imaging in detecting microscopic tumor within the treated primary tumor bed after neoadjuvant treatment (7). However, there is limited evidence for the role of DW imaging for EMVI and tumor deposit assessment after neoadjuvant therapy. Given recent data indicating a worse prognosis in patients with persistent EMVI at both postneoadjuvant MRI and pathology (8,9) and the known advantages of DW imaging over T2-weighted imaging alone for tumor detection in the primary tumor bed (10), it is important to evaluate DW imaging in this setting using correlation with a robust reference standard. Therefore, the primary aim of this study was to evaluate DW imaging in patients with LARC in the detection of viable EMVI and tumor deposit after neoadjuvant therapy with point-by-point comparison between imaging and pathology, with whole-mount pathology as the reference standard. The secondary aim was to evaluate whether DW assessment on postneoadjuvant MRI scans of EMVI and tumor deposit were associated with patient outcomes.

## Materials and Methods

### Ethical Approval

This retrospective single-institution study was approved by the institutional review board, with a waiver for written informed consent, and was compliant with the Health and Insurance Portability and Accountability Act.

### Study Sample

**Figure 1:**
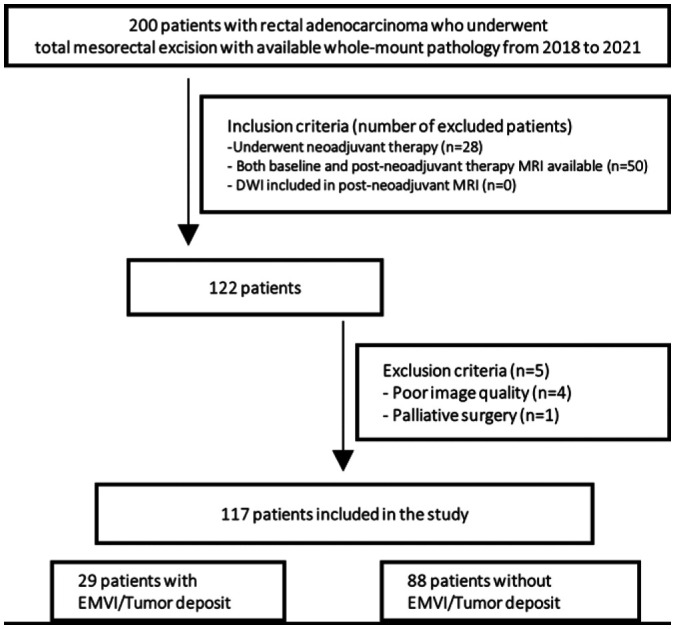
Flowchart of patient inclusion and exclusion criteria. DWI = diffusion-weighted imaging, EMVI = extramural venous invasion.

The study sample comprised consecutive patients with rectal adenocarcinoma who underwent total mesorectal excision with available whole-mount pathology at Memorial Sloan-Kettering Cancer Center institution from January 2018 to August 2021. Patients who underwent a watch-and-wait protocol or local surgery after neoadjuvant therapy were not included. The inclusion criteria were as follows: *(a)* patients who underwent neoadjuvant therapy, *(b)* availability of both baseline and postneoadjuvant MRI scans, and *(c)* DW imaging included in postneoadjuvant MRI. The exclusion criteria were as follows: *(a)* interval between postneoadjuvant MRI and surgery of more than 3 months, *(b)* poor image quality, and *(c)* palliative surgery (Fig 1).

### MRI Protocol

All patients underwent MRI performed with a 1.5- or 3.0-T scanner (Discovery MR750, Optima MR450w, Signa EXCITE, or Signa HDxt; GE Healthcare) with a phased-array coil. Two separate DW acquisitions were performed using two different *b* values (*b* value = 0 and 800 sec/mm^2^; *b* value = 0 and 1500 sec/mm^2^). All DW images were acquired in the axial plane with 5-mm section thickness. Details of DW image acquisition are summarized in Appendix S1 and Table S1.

### Evaluation of MRI for EMVI and Tumor Deposit

**Figure 2:**
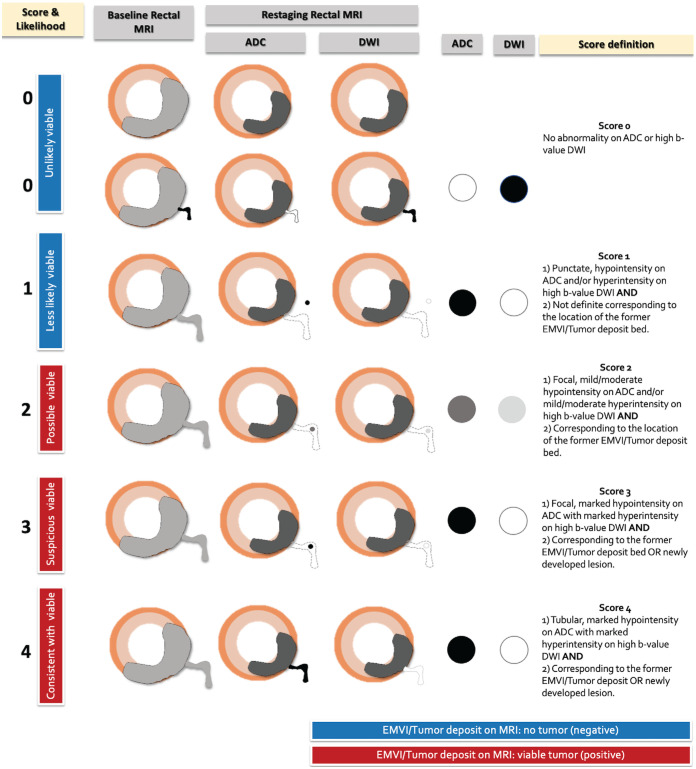
Diagram of the five-point Likert scale scoring system used to assess extramural venous invasion (EMVI) and tumor deposit on diffusion-weighted images with apparent diffusion coefficient (ADC) mapping. DWI = diffusion-weighted imaging.

Two readers (T.H.K. and N.H., with 1 and 5 years of experience in rectal MRI, respectively) independently reviewed baseline and postneoadjuvant MRI examinations to assess the presence of tumor deposit and EMVI. Tumor deposit was defined as irregular nodules within the mesorectum that were discontinuous from the primary tumor (1), and EMVI was defined as irregular serpentine tumor originating from the primary tumor on T2-weighted images. A five-point Likert scale was developed using standardized lexicon-associated numeric estimates of certainty (11) to estimate the likelihood of viable EMVI and tumor deposit on DW images and apparent diffusion coefficient on postneoadjuvant therapy MRI scans. EMVI and tumor deposit were assessed as a single entity using the following scale: 0, unlikely viable tumor (<10%); 1, less likely viable tumor (approximately 25%); 2, possibly viable tumor (approximately 50%); 3, suspicious for viable tumor (approximately 75%); and 4, consistent with viable tumor (more than 90%). The definitions and a diagram of the five-point DW imaging Likert scale are summarized in Table S2 and Figure 2.

Before applying the five-point Likert scale, two readers evaluated the presence of tumor deposit and EMVI on T2-weighted images on postneoadjuvant MRI scans, with a correlation to the baseline MRI scan. Then, the five-point Likert scale was used to assess four quadrants per patient for EMVI and tumor deposit, using the clockwise location on axial images, as follows: 12 to 3 o’clock, 3 to 6 o’clock, 6 to 9 o’clock, and 9 to 12 o’clock quadrants on both 800 and 1500 sec/mm^2^ images. When different scores were allocated on 800 and 1500 sec/mm^2^ images, the higher score was chosen. If there were no suspicious findings in the quadrant, a score of 0 was assigned. The final score was determined by consensus to assess the diagnostic accuracy of the five-point Likert scale. A radiologic evaluation using the baseline MRI, post-neoadjuvant MRI, and DW sequences was performed in the same session.

### Reference Standard

The reference standard was whole-mount pathology, allowing for a precise point-by-point correlation between MRI and whole-mount pathology. Details of whole-mount pathology processing are described in Appendix S1. One gastrointestinal pathologist (C.F., with 6 years of experience) reviewed the pathology slides with one radiologist (T.H.K.) to correlate EMVI and tumor deposit location in the four quadrants in each patient. For equivocal pathology slides, an experienced gastrointestinal pathologist (J.S., with 22 years of experience) was consulted, and final determinations were based on consensus. At pathologic analysis, tumor deposit was defined as tumor focus containing adenocarcinoma in the mesocolon or mesorectum, without histologic evidence of residual lymph node tissue, and EMVI was defined as a tumor within blood vessels located beyond the muscularis propria of the rectal wall (12).

### Statistical Analyses

Student *t* test and Pearson χ^2^ test were performed to find any significant associations between demographic or clinical characteristics and pathologic EMVI and tumor deposit status. Interreader agreement was evaluated between the two radiologists’ per-quadrant DW imaging five-point Likert scale scores using the weighted κ value with quadratic weights.

The diagnostic accuracy of the DW imaging five-point Likert scale in the detection of viable EMVI and tumor deposit on MRI scans was assessed on both per-quadrant and per-patient levels, using the optimal cutoff score determined with receiver operating characteristic curve analysis. The Fisher exact test was used to identify factors that were different between patients with false-negative findings and those with true-positive findings.

Logistic regression and survival analyses were performed using the optimal cutoff score. Logistic regression analysis was performed to predict pathologic EMVI and tumor deposit using postneoadjuvant MRI assessment and pathologic T and N stages. Kaplan-Meier curves and Cox proportional hazard models were used to compare disease-free survival and overall survival for positive versus negative cohorts based on postneoadjuvant MRI and pathologic EMVI and tumor deposit, respectively.

Details of each statistical analysis are described in Appendix S1. Statistical analyses were performed (J.Z. and M.C., with 18 and 23 years of experience, respectively) using R statistical software (version 4.1.3; The R Foundation for Statistical Computing), with *P* < .05 indicating a significant difference.

## Results

### Demographic and Clinicopathologic Characteristics

Of the 200 patients with rectal adenocarcinoma who underwent total mesorectal excision with available whole-mount pathology specimens, 50 patients were excluded due to the lack of either baseline or postneoadjuvant therapy MRI studies, and 28 patients were excluded due to the lack of neoadjuvant treatment. Additionally, four patients were excluded due to poor image quality, and one was excluded due to palliative surgery. Thus, a total of 117 patients (mean age, 56 years ± 12 [SD]; 70 male, 47 female) were included. The majority of included patients underwent total neoadjuvant therapy (100 of 117 patients [85%]; 29 with induction chemotherapy, 71 with consolidation chemotherapy). Thirteen of 117 patients (11%) underwent only chemotherapy, and four of 117 patients (3%) underwent standard neoadjuvant chemoradiation. The chemotherapy regimens used in total neoadjuvant therapy included fluorouracil, leucovorin, and oxaliplatin (mFOLFOX), capecitabine and oxaliplatin (CAPOX), or fluorouracil, leucovorin, and oxaliplatin (FLOX). Standard chemoradiation involved long-course radiation therapy (45 Gy in 1.8-Gy fractions to the pelvis, with a 5.4-Gy boost to the tumor) with concurrent fluorouracil or capecitabine. On baseline rectal MRI scans, 53 of 117 patients (45%) showed tumors in the midrectum, and 107 of 177 patients (91%) had nonmucinous tumors. The majority of tumors were clinically characterized as stage T3 or T4 (108 of 117 patients [92%]) and N positive (94 of 117 patients [80%]) at baseline. On the final pathologic assessment, viable EMVI and tumor deposit on pathology specimens were detected in 29 of 117 patients (25%) and in 44 of 468 quadrants (9.4%). Patients with viable EMVI and tumor deposit at pathology had higher pathologic T and N stage than those without (*P* < .001 for both). Table 1 summarizes the demographic and clinical characteristics of patients with and those without EMVI and tumor deposit.

**Table 1:**
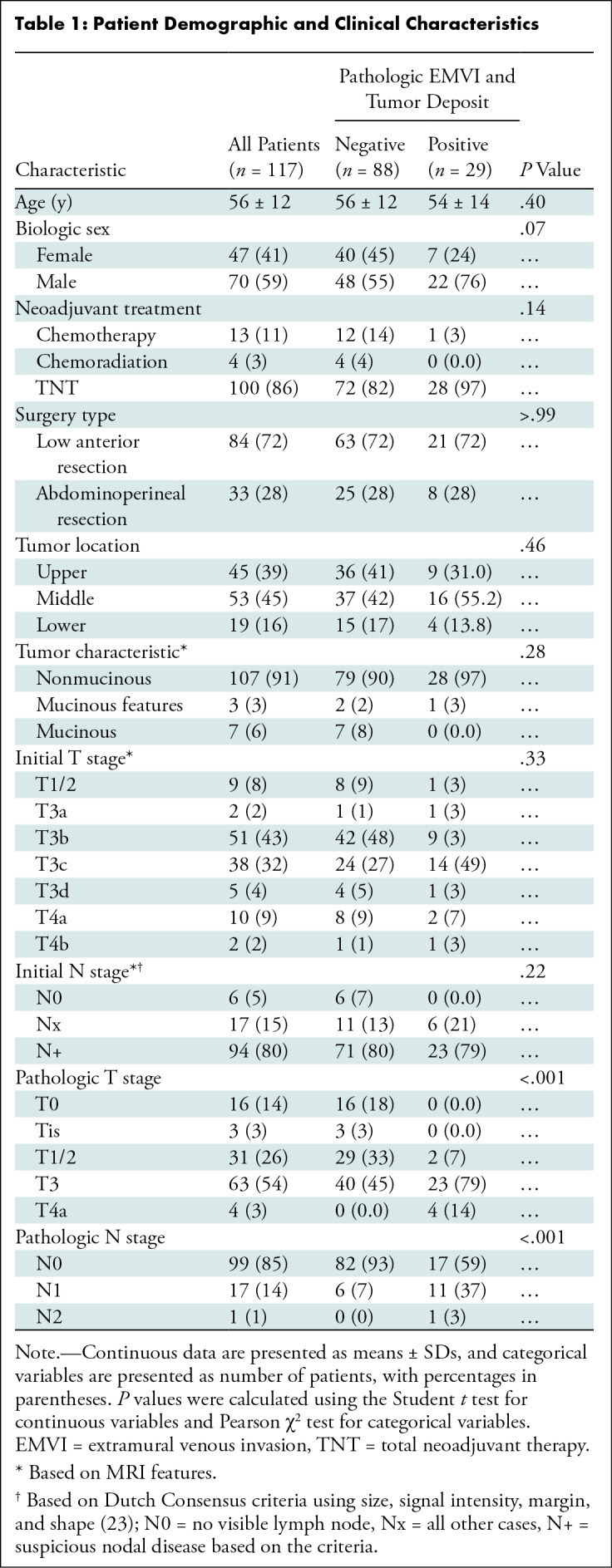
Patient Demographic and Clinical Characteristics

### Interreader Agreement and Diagnostic Accuracy of the DW Imaging Five-Point Likert Scale

Interreader agreement between the two radiologists using the five-point Likert scale on postneoadjuvant therapy MRI scans was good at the quadrant level, with a weighted κ value of 0.62 (95% CI: 0.51, 0.72).

The optimized cutoff value at the quadrant level for the five-point Likert scale was two, which had an area under the receiver operating characteristics curve of 0.76 (95% CI: 0.6, 0.84). Furthermore, the sensitivity, specificity, positive predictive value, and negative predictive value were 55% (95% CI: 41, 68), 96% (95% CI: 92, 99), 56% (95 CI: 34, 78), and 95% (95% CI: 93, 97), respectively. At the patient level, the optimized cutoff value of two had a sensitivity, specificity, positive predictive value, and negative predictive value of 62% (95% CI: 42, 79), 93% (95% CI: 86, 98), 75% (95% CI: 53, 90), and 88% (95% CI: 80, 94), respectively. Table 2 summarizes the diagnostic performance of the five-point Likert scale at both the quadrant level and the patient level.

**Table 2:**
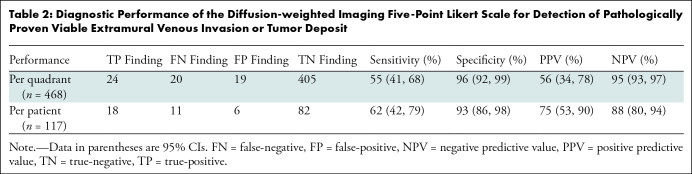
Diagnostic Performance of the Diffusion-weighted Imaging Five-Point Likert Scale for Detection of Pathologically Proven Viable Extramural Venous Invasion or Tumor Deposit

**Figure 3:**
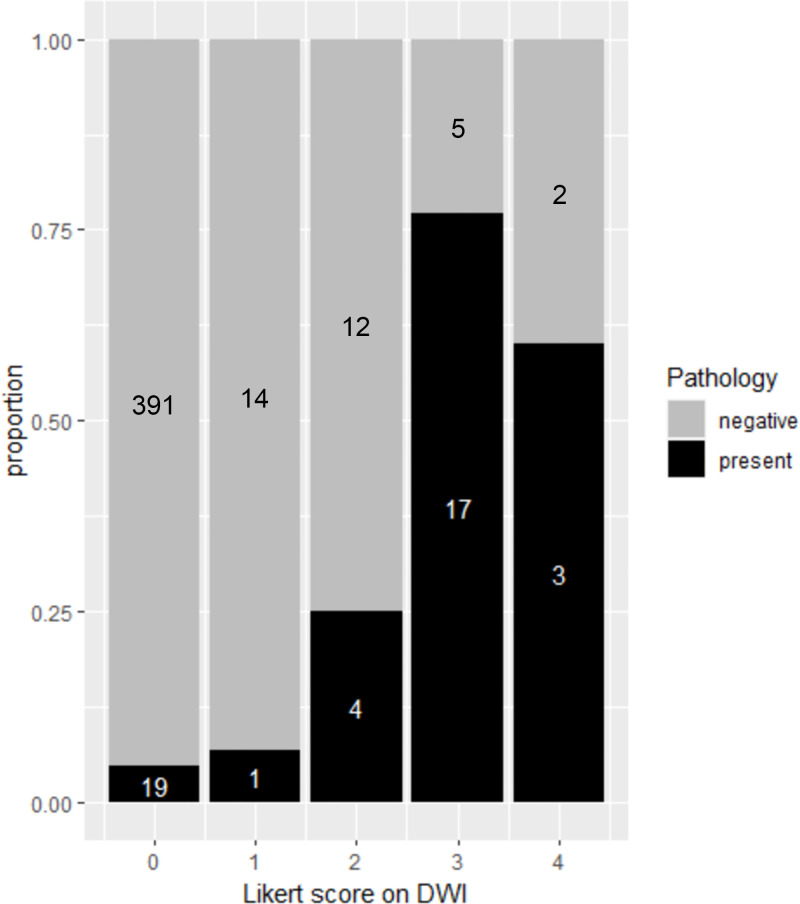
Stacked bar plot shows the per-quadrant distribution of extramural venous invasion and tumor deposit as assessed with the diffusion-weighted imaging (DWI) five-point Likert scale and whole-mount pathology. Gray area of the stacked bar represents quadrants with nonviable cells at pathology, and the black area of the stacked bars represents quadrants with viable cells at pathology. A total of 410 quadrants were assigned a Likert score of 0 (unlikely viable), 15 quadrants were assigned a Likert score of 1 (less likely viable), 16 quadrants were assigned a Likert score of 2 (possibly viable), 22 quadrants were assigned a Likert score of 3 (suspicious viable), and five quadrants were assigned a Likert score of 4 (consistent with viable).

**Figure 4:**
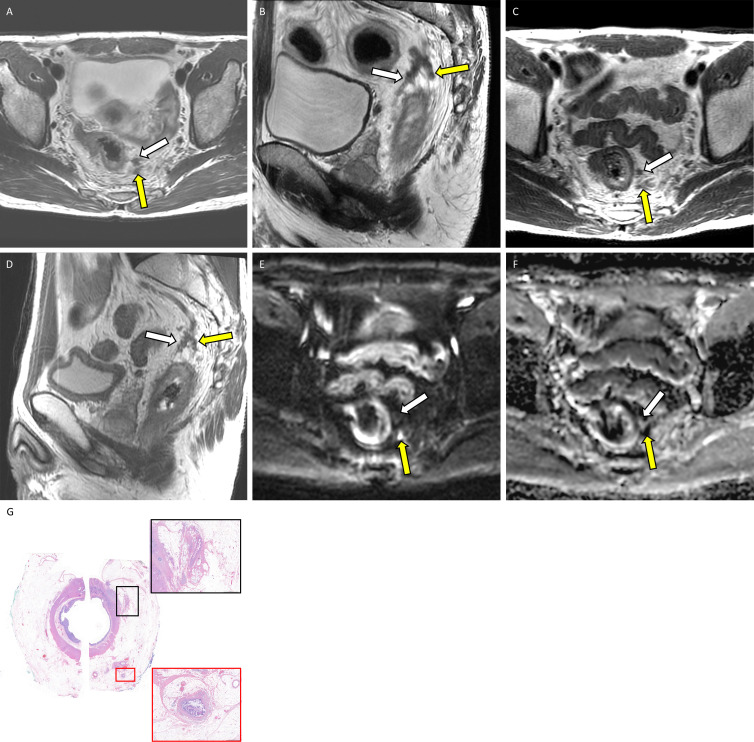
Example application of the diffusion-weighted (DW) image five-point Likert scale for assessing extramural venous invasion (EMVI) or tumor deposit at MRI with corresponding whole-mount pathology in a 46-year-old man with locally advanced rectal adenocarcinoma. Baseline **(A)** axial and **(B)** sagittal T2-weighted MRI scans show EMVI and tumor deposit in the 12 to 3 o’clock quadrant (white arrow) and in the 3 to 6 o’clock quadrant (yellow arrow). Postneoadjuvant **(C)** axial and **(D)** sagittal T2-weighted MRI scans show regressed EMVI and tumor deposit in both the 12 to 3 o’clock quadrant (white arrow) and the 3 to 6 o’clock quadrant (yellow arrow). Postneoadjuvant therapy **(E)** axial DW image and **(F)** apparent diffusion coefficient map yielded a score of 0 (unlikely viable) in the 12 to 3 o’clock quadrant (white arrow) and a score of 3 (suspicious viable) in the 3 to 6 o’clock quadrant (yellow arrow). **(G)** Photomicrograph (hematoxylin-eosin stain; original magnification, ×2) shows acellular mucin corresponding to the 12 to 3 o’clock quadrant, consistent with posttreatment change without viable malignant cells (black box; magnification, ×50). On the contrary, there is viable EMVI corresponding to the 3 to 6 o’clock quadrant (red box; magnification, ×50).

**Figure 5:**
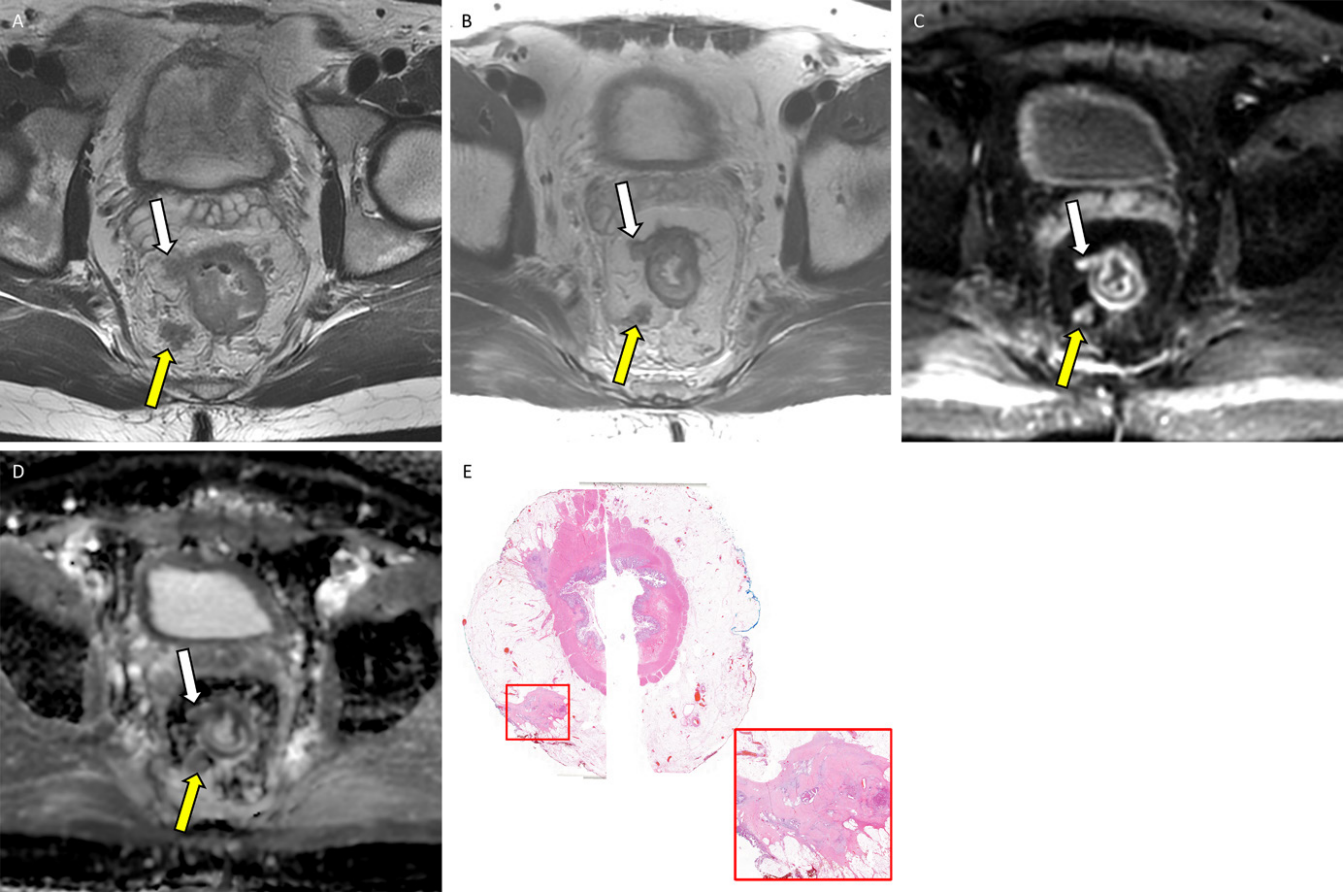
Example application of the diffusion-weighted (DW) image five-point Likert scale for assessing extramural venous invasion (EMVI) or tumor deposit on MRI scans with corresponding whole-mount pathology in a 36-year-old man with locally advanced rectal adenocarcinoma. **(A)** Baseline axial T2-weighted MRI scan shows EMVI and tumor deposit in the 6 to 9 o’clock quadrant (yellow arrow) and 9 to 12 o’clock quadrant (white arrow). **(B)** Postneoadjuvant therapy axial T2-weighted MRI scan shows partially regressed EMVI and tumor deposit in the 6 to 9 o’clock quadrant (yellow arrow) and 9–12 o’clock quadrant (white arrow). Postneoadjuvant therapy **(C)** axial DW image and **(D)** apparent diffusion coefficient map with a score of 4 (consistent with viable) in the 6 to 9 o’clock quadrant (yellow arrow) and in the 9 to 12 o’clock quadrant (white arrow). **(E)** Photomicrograph (hematoxylin-eosin stain, original magnification, ×2) shows viable tumor deposits corresponding to the 6 to 9 o’clock quadrant and 9 to 12 o’clock quadrant (red box; original magnification, ×50). Note that on **A** the lesion in the 9–12 o’clock quadrant presented similar to EMVI, contiguous from primary tumor bed; however, these turned out to be tumor deposits separate from primary tumor bed on **E**.

Of the 468 quadrants assessed, a Likert score of 0, 1, 2, 3, or 4 was assigned to 410 quadrants (88%), 15 quadrants (3%), 16 quadrants (3%), 22 quadrants (5%), and five quadrants (1%), respectively. Pathologically confirmed viable EMVI and tumor deposit were observed in 4.6% (19 of 410) of quadrants with a Likert score of 0, 6.6% (one of 15) of quadrants with a Likert score of 1, 25.0% (four of 16) of quadrants with a Likert score of 2, 77.2% (17 of 22) of quadrants with a Likert score of 3, and 60.0% (three of five) quadrants with a Likert score of 4 (Fig 3). Examples of the application of the five-point Likert scale to MRI scans and corresponding whole-mount pathology samples are presented in Figures 4, 5, and S1.

Of the 29 patients with pathologically proven viable EMVI and tumor deposit, MRI evaluation deemed 18 (62%) patients had findings positive for EMVI and tumor deposits (ie, true-positive findings) and 11 (38%) patients had findings negative for EMVI and tumor deposits (ie, false-negative findings). Comparative analysis showed that the presence of an upper rectal tumor was more common among patients within the false-negative group (six of 11 patients [55%]) than among patients with true-positive findings (three of 18 patients [17%]; Fisher exact test, *P* = .04). No other factors were found to differ between the false-negative and true-positive groups (Table 3).

**Table 3:**
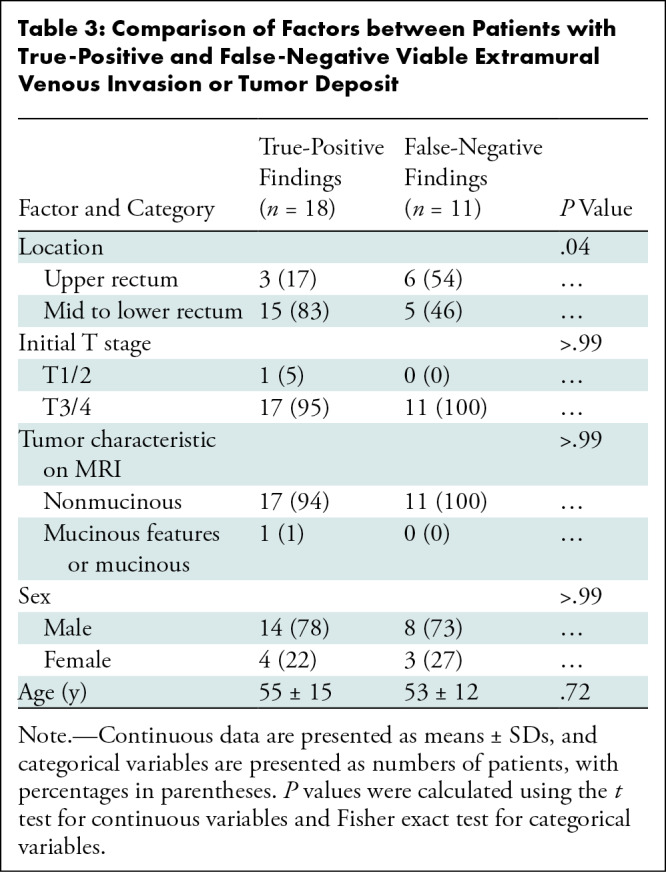
Comparison of Factors between Patients with True-Positive and False-Negative Viable Extramural Venous Invasion or Tumor Deposit

### Logistic Regression and Survival Analyses of DW Imaging Five-Point Likert Scale

The logistic regression model for predicting pathologic EMVI and tumor deposit with postneoadjuvant MRI assessment and pathologic T and N stages estimated that postneoadjuvant MRI assessment was significantly associated with pathologic EMVI and tumor deposit, as well as pathologic T and N stage (Table S3), indicating DW findings on postneoadjuvant MRI as an independent predictor for pathologic EMVI and tumor deposit, aside from pathologic T and N stage.

**Figure 6:**
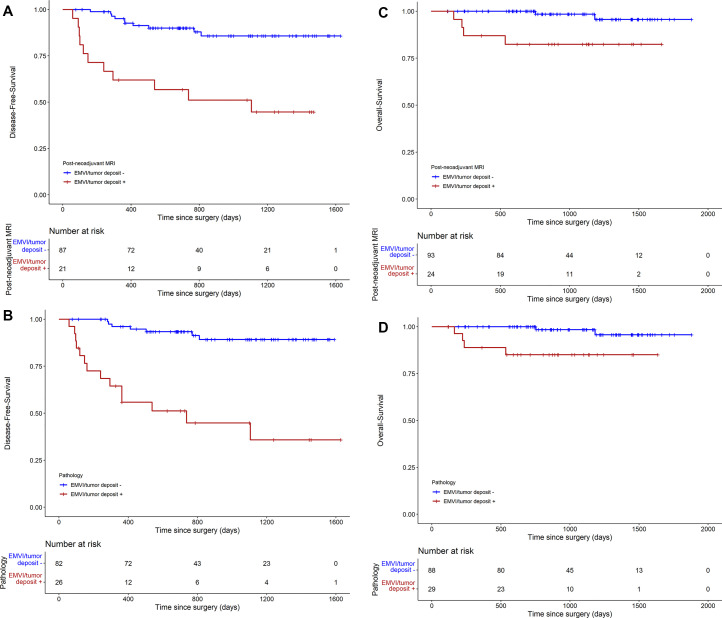
Survival analyses using the Kaplan-Meier curve. **(A)** Disease-free survival based on extramural venous invasion (EMVI) and tumor deposit at postneoadjuvant MRI, and **(B)** disease-free survival based on EMVI and tumor deposit at pathology. Survival analyses using the Kaplan-Meier curve. **(C)** Overall survival based on EMVI and tumor deposit at postneoadjuvant MRI, and **(D)** overall survival based on EMVI and tumor deposit at pathology.

During median follow-up of 985 days, a total of 21 patients developed recurrence (20 patients developed distant metastasis, one patient developed a local recurrence). In the survival analyses, not only did patients with EMVI and tumor deposit on final pathology specimens have significantly shorter disease-free survival (HR, 9.41; 95% CI: 3.78, 23.45; *P* < .001) and overall survival (HR, 7.88; 95% CI: 1.42, 43.75; *P* = .005) than those without, patients with EMVI and tumor deposit on postneoadjuvant MRI scans also had significantly shorter disease-free survival (HR, 5.64; 95% CI: 2.39, 13.32; *P* < .001) and overall survival (HR, 8.85; 95% CI: 1.61, 48.49; *P* = .002) than those without (Fig 6).

## Discussion

Extramural venous invasion (EMVI) and tumor deposit are recognized as poor prognosticators in patients with locally advanced rectal cancer (LARC), and prediction of viable EMVI and tumor deposit on MRI scans after neoadjuvant therapy is becoming clinically important. We evaluated the diagnostic performance of diffusion-weighted (DW) imaging for detection of viable EMVI and tumor deposit in patients with LARC after neoadjuvant therapy. Using whole-mount pathology as the reference standard, DW imaging assessment based on a five-point Likert scale showed specificity of 96% and sensitivity of 55% with good interreader agreement (κ = 0.62). The high specificity indicates that positive findings on DW images are meaningful and are rarely false positive. On the other hand, the moderate sensitivity reflects the inherent limitation of imaging to detect microscopic EMVI and tumor deposit. Furthermore, the presence of positive EMVI and tumor deposit on postneoadjuvant MRI scans using the Likert scale was a significantly poor prognosticator.

Previous studies have evaluated the diagnostic accuracy of MRI in the detection of EMVI and tumor deposit, mostly based on T2-weighted images without direct radiologic-pathologic correlation (13–16). In a retrospective study of 79 patients, Ahn et al (17) reported no added value of DW imaging compared with T2-weighted imaging alone for the detection of EMVI in patients with rectal cancer who underwent primary tumor resection without neoadjuvant therapy. On the other hand, Fornell-Perez et al (18) reported the improved diagnostic performance with the addition of DW imaging, particularly in 46 patients after chemoradiation. While these two studies used a binary assessment (ie, present or absent) to detect EMVI on DW images, our study developed and used a five-point scale DW imaging scoring system to assess the viability of EMVI and tumor deposit, which took into consideration not only the signal intensities on DW images and apparent diffusion coefficient but also the location of the findings, such that the findings on the postneoadjuvant MRI study could be correlated with those on the baseline MRI study. The new scoring system used in our study achieved a sensitivity and specificity of 62% and 93%, respectively, for detection of viable EMVI and tumor deposit based on a per-patient analysis.

Pathologic EMVI and tumor deposit have been consistently reported to be worse prognostic factors (19,20). However, as pathologic samples of EMVI and tumor deposit can be obtained only after surgery, EMVI and tumor deposit on MRI studies have been widely accepted as surrogate markers for predicting a worse prognosis, which was based on the premise that EMVI and tumor deposit on MRI scans correspond to pathologic EMVI and tumor deposit. Our results not only corroborate those of previous studies regarding EMVI and tumor deposit on MRI scans related to worse prognosis (21) but also prove the premise to be true using a rigorous method with whole-mount pathology. Given increasing numbers of patients who undergo a watch-and-wait approach after neoadjuvant therapy, patients with a positive finding for EMVI and tumor deposit on DW images at postneoadjuvant MRI may warrant individualized treatment planning.

Interestingly, the proportion of quadrants with pathologically proven viable EMVI and tumor deposit was higher in patients with a score of 3 (17 of 22 patients [77%]) than in those with a score of 4 (three of five patients [60%]), partly due to the low incidence of quadrants with a score of 4. It is also noteworthy that DW imaging is prone to artifacts from things such as air interface magnetic susceptibility or motion. For instance, in our study, false-negative findings were more likely to be present in patients with an upper rectal tumor.

In our study, EMVI and tumor deposit were assessed as a single entity for the following reasons. First, pathologic differentiation between EMVI and tumor deposit is difficult among pathologists (22). Second, there has been increasing recognition that EMVI on MRI scans is not always consistent with EMVI on whole-mount pathology specimens based on studies evaluating EMVI on T2-weighted images (5,6). Additionally, tumor deposits on postneoadjuvant MRI scans were often difficult to differentiate from lymph nodes. This required correlation with the baseline MRI study, in which tumor deposits were more apparent.

This study had some limitations. First, this was a retrospective single-center study; therefore, studies at other institutions are necessary to validate our findings. Second, the diagnostic accuracy of T2-weighted imaging to detect EMVI and tumor deposits was not evaluated separately. T2-weighted imaging was used only to identify EMVI and tumor deposits to differentiate them from a normal vessel. Once EMVI and tumor deposit were identified, the innovative aspect of this investigation was to interrogate DW imaging for signal indicating viable tumor, in much the same way as paired T2-weighted imaging and DW imaging sequences are used to analyze the primary tumor bed. Since fibrosis on T2-weighted images has limited accuracy for concurrent viable tumor in the primary tumor bed, we made the same assumption for EMVI and tumor deposit and thus took it a step further and focused on DW imaging only. Third, the five-point Likert scale is a qualitative assessment. Interreader agreement in multiple readers with variable experience in interpreting rectal MRI is required to validate the Likert scale. Fourth, quantitative apparent diffusion coefficient assessment was not assessed in the current study. Fifth, we used the same data to determine the optimal cutoff on the Likert scale and to estimate sensitivity and specificity. The lack of a validation set and the application of the optimal cutoff of the Likert scale to the same data would lead to an overestimation of diagnostic performance. Further studies are required to validate the diagnostic performance of the Likert scale. Sixth, our study aim was to correlate MRI and pathology findings, so we excluded patients with LARC who were undergoing a watch-and-wait protocol; therefore, we may have selected patients with higher risk.

In conclusion, the evaluation of diffusion-weighted images using a five-point Likert certainty scale showed high specificity and moderate sensitivity in the assessment of viable extramural venous invasion (EMVI) and tumor deposits in patients with locally advanced rectal cancer after neoadjuvant therapy. The presence of viable EMVI and tumor deposit based on the Likert certainty scale on postneoadjuvant MRI was associated with a worse prognosis. Further studies with a prospective design are needed for the validation of the observations in the current study.
